# Positive imagery in depressive suicidal patients: A randomized controlled trial of the effect of viewing loved ones’ photos on mood states and suicidal ideation

**DOI:** 10.1016/j.heliyon.2023.e22312

**Published:** 2023-11-14

**Authors:** Fahimeh Alsadat Hosseini, Maryam Shaygan, Zahra Jahandideh

**Affiliations:** a Community Based Psychiatric Care Research Center, School of Nursing and Midwifery, Shiraz University of Medical Sciences, Shiraz, Iran; b School of Nursing and Midwifery, Shiraz University of Medical Sciences, Shiraz, Iran

**Keywords:** Major depressive disorder, Mood states, Positive imagery, Suicidal ideation

## Abstract

According to research, it has been suggested that individuals who are affected by depression could potentially engage in the creation and experience emotional advantages relating to positive events directed towards the past or future, with the condition that they are provided with suitable mental imagery techniques. The main aim of this study was to assess the impact of utilizing positive imagery, specifically through the utilization of photographs featuring loved ones, on mood states and suicidal ideation among individuals diagnosed with depression and exhibiting suicidal tendencies. This randomized, double-blind, controlled crossover trial was conducted among 78 hospitalized depressive patients at three psychiatric services between April and August 2019. The patients participated in four individual picture-viewing sessions on four consecutive days. The four categories of pictures were included: loved ones, neutral faces of strangers, natural landscapes, and optical illusions. Directly prior to and immediately following the observation of the visual stimuli (photographs), the Brunel Mood Scale (BRUMS) and the Beck Scale for Suicide Ideation (BSSI) were completed by the patients. Repeated measures ANOVAs conducted in this study revealed a significant main effect of time on ratings of tension, depression, fatigue, vigor, calmness, and happiness (P values < 0.001). Additionally, statistically significant interactions were identified between picture category and time in relation to the variables of tension, depression, fatigue, vigor, calmness, and happiness (P values < 0.001). The analysis did not reveal a significant main effect of time on ratings of anger, confusion, and suicidal ideation (P values > 0.05). Likewise, the interaction between picture category and time did not yield significant results for the variables of anger, confusion, and suicidal ideation (P values > 0.05). The positive imagery procedure using the presentation of loved ones’ photos showed beneficial effects on the mood states of depressed patients. The findings of this study suggest that incorporating a greater emphasis on positive imagery within the context of clinical depression may offer potential advantages. This highlights the potential for novel opportunities in the treatment of depression.

**Trial registration:**

The study has been registered in the Iranian Registry of Clinical Trials (registration number: IRCT20180808040744N1; first registration date: December 22, 2018; website: https://en.irct.ir/trial/33186).

## 1 Introduction Background

Major Depressive Disorder (MDD), as defined in the DSM-5 (Diagnostic and Statistical Manual of Mental Disorders, Fifth Edition), is a common mental health condition distinguished by the occurrence of one or multiple major depressive episodes. These episodes involve persistent feelings of sadness, reduced interest or pleasure in activities, changes in appetite or weight, disturbances in sleep patterns, fatigue, feelings of worthlessness or excessive guilt, difficulties in concentration, and recurrent thoughts of death or suicide [1]. In fact, the presence of suicidal tendencies is prevalent and considered the most significant manifestation of major depressive disorder [2]. The occurrence of suicidal thoughts serves as a precursor to suicide attempts and plays a substantial role in completed suicide cases among individuals diagnosed with MDD [3,4].

According to the positive attenuate view, depression may attenuate access to and/or use of positive emotional resources that may aid recovery. Thus, depressed individuals have difficulties mending their sad mood with happy memories [5]. There is a proposition that depression may be linked to difficulties or limitations in the ability to generate mental imagery [6]. In accordance with the subcomponents model of depression, it is postulated that depressed mood is not solely sustained and intensified by negative imagery processes but also by reduced efficacy in generating positive mental imagery [7]. In a study conducted by Holmes and colleagues, involving qualitative interviews with a cohort of 15 individuals who had experienced depression and suicidal tendencies, it was observed that all participants reported the presence of mental imagery associated with future suicide, which demonstrated a significant association with suicidal ideation [8]. Hence, mental images could hold particular significance in affective disorders and exert a substantial influence on the characteristics of depression. However, the empirical research studying the impact of mental imagery on emotions and clinical symptoms in depression remains limited and subject to controversy.

The existing literature provides extensive confirmation of the capacity of mental imagery to elicit intense emotional responses [9,10]. Imagining positive events can induce stronger positive effects than thinking about them verbally [11]. Previous studies have established that selectively impoverished positive (compared to negative) imagery in depressed individuals might have a significant impact on their ability to recall their past positive memories or imagine a positive future (for a review, see Holmes et al., 2016) [12,13]. The literature provides clear evidence regarding the effectiveness of using the recall of positive autobiographical memories as a strategy to generate positive mental imagery and induce positive emotional states [14]. This approach redirects individuals' focus towards the positive emotions linked to their autobiographical memories while also stimulating the exploration, expansion, and processing of positive personal meaning that can potentially counteract negative beliefs [15]. In the context of positive memory recall in current or recovered depression, Werner-Seidler & Moulds showed that participants who were instructed to focus on the concrete, sensory aspects of their pleasant memories could emotionally benefit from the recall of positive memories regardless of a depressive condition (depressed or recovered from depression), while the abstract processing of pleasant memories could not improve mood [16]. These findings indicate that individuals with depression may be able to generate and emotionally benefit from the imagery of past- or future-directed positive events if given appropriate mental imagery. However, previous studies focusing on the use of mental imagery for individuals with suicidal ideation have primarily examined the impact of mental images related to suicide [12,[17], [18], [19]], whereas the effect of positive mental imagery through the utilization of positive memories on depressive suicidal patients has been investigated in a solitary study conducted by Knagg et al. (2022) [20]. The study implemented a concise relaxation technique, followed by guided visualization of a positive memory, with the primary objective of addressing suicidal ideation among university students. Additionally, participants were encouraged to contemplate their understanding and evaluation of the situation that led to the experience of positive emotions. The outcomes of this preliminary investigation exhibited encouraging indications regarding the practicality and acceptance of the positive mental imagery intervention among students who were contending with suicidal ideation [20]. Then, it is not surprising that positive mental imagery utilizing the recall of positive memories may emerge as a promising and innovative treatment approach for depressive suicidal patients. However, it has been relatively overlooked in the treatment of depressive suicidal patients, and the study of mental imagery for these patients is still in its early stages of research.

The technique of recollecting positive autobiographical memories presents a valuable opportunity for cultivating a more well-rounded outlook by directing attention towards positive experiences [21]. Over time, these positive reflections can gradually become integrated into an individual's self-perception and overarching perspective, resulting in transformative effects by establishing positive schemas. Moreover, this practice may foster the acquisition of emotional regulation skills, heightening one's understanding of the influence of attention and cognition on emotions, enhancing attentional control, and promoting hope sense [22] and connectedness [15]. These factors are critical in safeguarding against the exacerbation of depressive symptoms and suicidal thoughts [23]. Family photographs act as an important tool for remembering pleasant and memorable family moments (e.g., weddings, birthdays, trips, etc.) that evoke experiences of being loved [24]. Viewing photographs of loved ones can also create a sense of calmness, security, and support, which can be efficient in adapting to stressful situations and crises [25,26]. This may support the potential effect of viewing loved ones' photographs on boosting mental imagery of positive memories in depressed individuals with suicidal ideation. However, it is important to note that there is a lack of studies that have specifically assessed the effects of mental imagery using loved ones' photographs on depressive suicidal patients. So far, most studies examining the effect of viewing loved ones' pictures have focused on laboratory-induced pain [[27], [28], [29]]. For instance, Shaygan et al. (2017), in a clinical study conducted on patients with chronic pain, observed that the viewing of pleasant photographs, particularly images of loved ones, resulted in pain reduction among patients undergoing treatment at a multidisciplinary pain center [29]. Therefore, examining the effects of mental imagery using loved ones' photographs in depressive suicidal patients can provide new and valuable insights to develop some novel interventions for individuals struggling with depression and suicidal ideation.

Depressive suicidal patients constitute a highly vulnerable population confronted with the complexities of depression and self-harming ideation [30]. Individuals grappling with this condition often experience a surplus of intrusive and involuntary negative mental imagery, accompanied by a scarcity of positive mental imagery. Moreover, they may encounter difficulties in deliberately eliciting specific mental images associated with their personal past or future encounters [5,6]. Due to the significant impact of imagery, particularly via the recalling method of positive autobiographical memories, on emotional states, the use of imagery techniques related to recalling positive autobiographical memories, such as family photographs, appears to be a promising therapy option for depressive suicidal patients, which merits more investigation and development. Nevertheless, when it comes to managing depressive patients with suicidal tendencies, the utilization of this imagery technique and other innovative methods for fostering positive mental imagery remains largely underutilized [31,32]. Furthermore, inpatients diagnosed with Major Depressive Disorder (MDD) are often at an elevated risk for experiencing suicidal thoughts or ideation, self-harm, and re-admission, making them a particularly vulnerable population requiring immediate and intensive care. On the other hand, hospitalizations for patients with MDD impose a substantial burden on patients, their families, and the healthcare system. Therefore, there is a critical need for innovative treatment interventions that have the potential to mitigate these issues. By investigating the impact of positive imagery utilizing photos of loved ones on mood states and suicidal ideation among hospitalized depressive suicidal patients, our study offers a unique, innovative, and patient-centered approach to address the main issues of these patients. These findings may contribute to the development of more accessible and cost-effective adjunctive treatment strategies for patients experiencing the common, debilitating, and costly condition of major depressive disorder accompanied by suicidal ideation. Therefore, the aim of the current study was to examine the impact of positive imagery, specifically through viewing photographs of loved ones, on mood states and suicidal ideation among hospitalized patients with depression and suicidal tendencies. In this study, it is assumed that the imagery for past- or future-directed positive events (using the photographs of loved ones) has beneficial effects on improved mood states and reduced suicidal ideation in these patients.

## 2 Methods

**Fig. 1:**
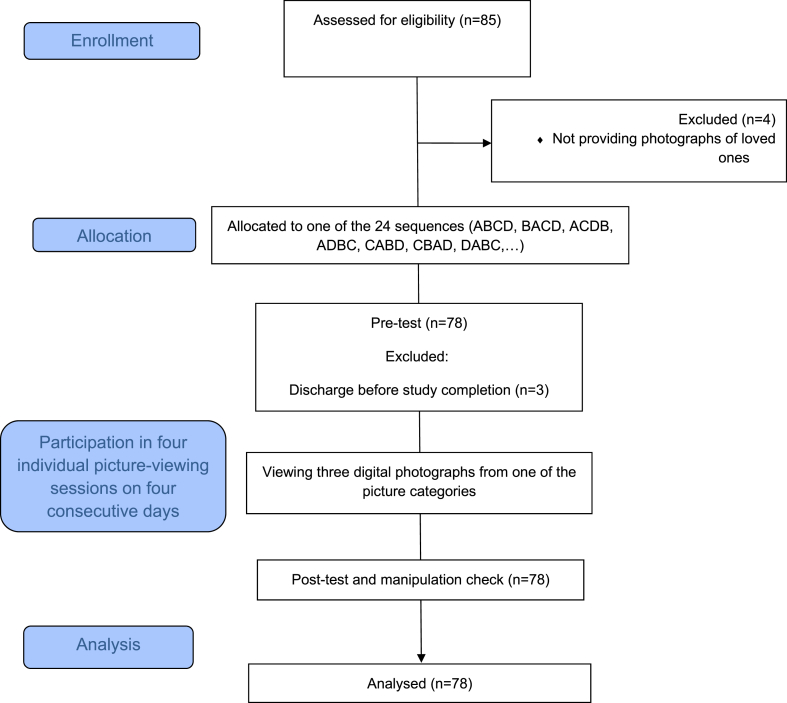
CONSORT flow diagram (crossover design).

This study has been conducted and reported in line with the CONSORT extension guidelines for randomized crossover trials [33], which provide guidelines for reporting randomized controlled trials with a crossover design in a transparent manner. The crossover randomized design allows for efficient comparison of interventions by utilizing each patient as their own control, reducing confounding factors and the required sample size. It also eliminates potential carryover effects and enables a precise assessment of treatment effects. Reporting this study in accordance with the CONSORT extension enhances the credibility and replicability of the study's findings and provides a comprehensive framework for accurately reporting essential aspects of study design, randomization, blinding, and data analysis. The CONSORT Flow Diagram of this study is shown in Fig. 1.

### 2.1 Procedure and design

• **Study design**

The crossover randomized design was used in this study because it could yield a more efficient comparison of different interventions (i.e., viewing different picture categories) than a parallel design. Importantly, the crossover design may substantially reduce the influence of confounding covariates and the number of patients needed because each crossover patient acts under his/her own control. Moreover, the disturbing effects of the patient's possible mood swings on different days were eliminated by examining the patient's mood states immediately preceding and following viewing the photos. Therefore, the crossover trial could remove from the intervention comparisons any components that may be related to the differences between the subjects [34]. This enhances the validity of intervention comparisons. Furthermore, some of the inherent disadvantages associated with crossover design, such as a potential carryover effect and a large dropout rate, were not expected in the present study.

• **Recruitment**

Patient recruitment was conducted between April and August 2019. Due to the increased occurrence of depression among women compared to men (2:1), we included a higher ratio of women to men (57 women, 28 men) in the sample. The eligible patients were recruited from consecutive inpatient admissions to the biggest psychiatric services in Shiraz, Iran. A psychiatric nurse (the third author) provided the patients with detailed information regarding the study's purpose and inquired about their willingness to participate in it.

• **Picture stimuli**

The patients were presented with four categories of pictures: images of their loved ones, neutral faces of strangers, natural landscapes, and optical illusions. It was assumed that each category would elicit emotional states with varying levels of valence and arousal.

Before the beginning of the experiment, patients were requested to provide three photos of their own choice of their loved one in their best memories with him or her (e.g., on a trip, at a party or birthday, etc.). The patients received instructions to provide three photos of a currently living loved one (such as a close current friend or family member, not a former significant other) in a specific positive event (for example, photos of the patient and his/her spouse on a honeymoon) in which the patient had felt a very positive mood. It was assumed that the inclusion of photographs depicting loved ones would elicit the most positive emotional response, evoking memories of joyful experiences with family and friends as well as reminding the patient of the positive aspects of life that may have been overlooked or forgotten [28,35]. As a control to test this assumption, we used three photographs of strangers with neutral faces within the category of "photographs featuring human beings," which was without the components of love and happy memories elicited through looking at the photos of loved ones. Three pictures of three strangers (two women and one man who were independent of the study and had no contact with the patients) were captured before the intervention within the laboratory setting, following standardized conditions. The participants who served as strangers in the study were provided with instructions to wear a dress in white, black, or grey color tones and maintain a direct gaze towards the camera, refraining from smiling [28]. All of the strangers' photographs were evaluated by two independent raters for deviations from neutral stimulation. When the strangers’ photographs were not evaluated as neutral, new photographs were taken. The two additional categories comprised pictures that did not feature a human context, namely photographs depicting natural landscapes and optical illusions. These pictures were taken from our previous study [29]. It was assumed that the landscape photographs would evoke a predominantly positive valence. However, it was noted that these images lacked the stimulating properties typically associated with stimuli that include humans, with whom the patient has shared positive memories. The optical illusions, such as the rabbit-duck illusion, were considered emotionally neutral stimuli. They were intended to serve as attentional controls, diverting attention away from a depressed mood. Patients were instructed to identify the various perceptual images within these pictures. All the pictures in our study were colored and were 2248 mm in length and 4000 mm in width.

• **Randomization**

The assignment of patients to specific picture categories during each session was determined randomly. The randomization process, determining the sequence of picture categories for each patient, was conducted prior to the commencement of the study sessions. An independent individual, not directly involved in the trial, employed a computerized randomization program for this purpose.

Randomization of picture categories in this study was performed to ensure unbiased allocation to each patient. By using a computerized randomization program and an independent person, the risk of systematic differences between groups was minimized. This rigorous approach enhances the internal validity of the study and allows for a more reliable evaluation of the effects of different picture categories.

• **Blinding**

Each patient was unaware of the sequence of photographs that he/she would see during the study sessions. The sequence of photographs for each patient was determined by an independent observer, not a team member in the current study (allocation concealment). In addition, the interpretation of questionnaires, data entry, and analysis were done by independent researchers not involved in this trial. The blinding design ensured that both the patients and the researchers administering the interventions were unaware of the sequence of photographs assigned to each patient. This blinding procedure minimized biases and increased the internal validity of the study by preventing conscious or subconscious influences on the assessment of outcomes.

### 2.2 Participants

• **Study sample and settings**

The sample for this study consisted of 78 patients who were admitted to three psychiatric services in Shiraz, located in the southern region of Iran. All participants in the study had a primary diagnosis of unipolar Major Depressive Disorder (MDD), as defined in the Diagnostic and Statistical Manual of Mental Disorders, Fifth Edition (DSM-V). The diagnoses were confirmed by a psychiatrist using the Structured Clinical Interview for DSM (SCID-5) [1]. The following inclusion criteria were used: over 18 years of age, ability and willingness of individuals to participate in the study, being literate, receiving an SSRI as antidepressant treatment (due to the widespread use of SSRIs as first-line treatment for depressive disorders and their effectiveness and established safety profile), having suicidal ideation determined by screening items of the Beck Scale for Suicide Ideation [36], and the screening questions in the CIDI 3.0 “Depression” module [37]. The study applied the following exclusion criteria: individuals diagnosed with substance abuse disorder, a history of bipolar disorder, the presence of psychotic symptoms, severe symptoms of depression such as mutism, specific phobias such as fear of strangers, receiving electroconvulsive therapy (ECT) during the study, receiving antidepressants other than selective serotonin reuptake inhibitors (SSRIs) during the study (for having a more homogeneous sample and reducing potential confounding variables), unwillingness or inability to provide photos of loved ones, and being unable or unwilling to continue participating in the study.

• **Sample Size Calculation**

The determination of the required sample size was according to having at least 99 % power (β = 0.01) and a 5 % significance level (α = 0.05) to detect a mean difference of 1.18 points (the “Minimal Difference change” (MDC)) in the Brunel Mood Scale (BRUMS) Score, which served as the primary outcome measure in the current study. Based on a between-subject standard deviation of 1 [38], assuming a two-sided test on data from a two-period, four-sequence (Balaam) cross-over design (2 × 4) and 20 % attrition, a total of 85 patients were needed to satisfy the same significance and power requirements. The PASS v21.0.3 software (NCSS LLC, Kaysville, UT, USA) was utilized for the calculation of the sample size in the study. To determine the effective sample size, the Minimal Detectable Change (MDC) was computed utilizing the subsequent formula: MDC = (s_baseline_ × √(1 – ICC)) × 1.96 × √2, where s_baseline_ corresponds to the standard deviation of a baseline test and ICC was considered as 0.88 [39,40].

• **Ethical considerations**

In the study, all patients who voluntarily agreed to take part were informed about the research project's details and their right to withdraw from the study if they wished to do so. Written informed consent was obtained from each participant. The data was collected anonymously, and questionnaires were coded by numbers. While viewing the photos, the patients' privacy was respected, and no one other than the patient and the psychiatric nurse could access the patients' loved ones' photos. Consent was obtained from all individuals depicted in the photographs. The study received ethical approval from the Ethics Committee of the Shiraz University of Medical Sciences (IR.SUMS.REC.1399.011). Additionally, the study was registered in the Iranian Registry of Clinical Trials (registration number: IRCT20180808040744N1; first registration date: December 22, 2018).

### 2.3 Intervention procedures

The eligible and interested patients were asked by the psychiatric nurse (third author) to provide three joint pictures of themselves with their loved ones (for example, a photograph of the patient in the hospital with his/her baby after birth) within two days after giving informed consent. Following the provision of photographs depicting loved ones, the patients engaged in a series of four individual picture-viewing sessions. These sessions occurred consecutively over four days and were conducted in a dedicated meeting room setting at 6 p.m. At the beginning of each session, patients were asked to sit in a comfortable chair in front of which there was only a blank white wall without any handwriting, pictures, or windows. After that, the patients were shown three digital photographs from one of the picture categories. The patients were requested to talk about each picture for 3 min while viewing it. While looking at the loved ones’ photographs, the patients were requested to explain the good memories evoked by the photos. The patient was asked to imagine himself/herself next to his/her loved one in that past memory. He/She was also asked to imagine a common positive mental image about both of them in the future and explain it in detail. As in the case of other photographs, such as photographs of strangers and landscapes, the patient was asked to describe his/her feelings while imagining himself/herself next to a stranger or on a landscape. In the case of optical illusions, the patient was asked to concentrate on the picture and find different visual images. The Brunel Mood Scale (BRUMS) and the Beck Scale for Suicide Ideation (BSSI) were completed by the patients directly prior to and immediately after the participants engaged in viewing the pictures. Furthermore, a manipulation check was conducted upon completion of the task.

### 2.4 Measures

In addition to assessing socio-demographic (age, sex, marital status, educational level) as well as psychiatric (e.g., history of depressive symptoms and the duration of hospital stay before the first study session) characteristics, the following outcome measures were applied.

### 2.5 Primary clinical outcome

Mood states were regarded as the primary clinical outcome, given the assumption that they act as a predictive factor of suicidal ideation in depressive patients [41,42]. Mood states were evaluated by the Brunel Mood Scale (BRUMS) [43]. This scale comprises 32 items that are rated on a 5-point Likert scale, spanning from 0 (not at all) to 4 (extremely). This scale evaluates eight dimensions of mood: mood; anger (items 9, 15, 25, and 29); confusion (items 3, 13, 23, and 32); depression (items 7, 8, 16, and 20); fatigue (items 4, 10, 14, and 28); tension (items 1, 17, 18, and 24); vigor (items 2, 19, 26, and 30); calmness (items 6, 12, 22, and 27); and happiness (items 5, 11, 21, and 31) [43]. In the evaluation of internal consistency, Lin et al. (2007) reported Cronbach's alpha coefficients for different factors as anger = 0.77, calmness = 0.71, confusion = 0.69, depression = 0.72, fatigue = 0.73, happiness = 0.88, tension = 0.72, and vigor = 0.72 [43].

In a study by Farrokhi et al. [40], the reliability (Cronbach's alpha; anger: 0.72; confusion: 0.72; depression: 0.70; fatigue: 0.76; tension: 0.74; vigor: 0.80; calmness: 0.86; happiness: 0.87) and construct validity of the Persian version of the instrument were confirmed. In the current study, the reliability of the questionnaire, estimated using Cronbach's alpha, was as follows: anger (α = 0.73), confusion (α = 0.71), depression (α = 0.76), fatigue (α = 0.70), tension (α = 0.76), vigor (α = 0.78), calmness (α = 0.74), and happiness (α = 0.81).

### 2.6 Secondary clinical outcome

Suicidal ideation (SI) was evaluated using the Beck Scale for Suicide Ideation (BSSI) [44]. The questionnaire, the Beck Scale for Suicide Ideation (BSSI), measures the existence and intensity of patients' thoughts, plans, and intentions related to suicide. Comprising 19 self-reported items, the BSSI includes five screening items that help identify individuals with sufficient suicidal ideation (scores 1 and 2) to warrant completion of the entire scale [45]. The items on the BSSI are rated on a three-point Likert scale, ranging from 0 to 2. The total score on the scale can range from 0 to 38. No specific cut-off point has been established for the scale. Higher scores on the BSSI indicate a greater level of suicidal risk [45].

The reliability and validity of the BSSI in the English language have been extensively examined. In most cases, the Cronbach's alpha coefficient exceeded 0.85, indicating high reliability. Furthermore, the BSSI scores demonstrated significant correlations with measures of depression, hopelessness, anxiety, history of suicide attempts, and future suicide attempts [46]. In a study conducted by Esfahani et al., the construct validity of the BSSI was evaluated by estimating the correlation between its total score and the Symptoms Checklist-90-Revised scale (SCL-90-R) (r = 0.50) [47]. In this study, the Cronbach's alpha coefficients for the screening section and the entire Persian version of the scale were found to be 0.83 and 0.84, respectively [47]. In the present study, for the screening segment of the Persian version of the scale, the Cronbach's alpha coefficient was calculated to be 0.85, while the overall scale exhibited a coefficient of 0.80.

### 2.7 Manipulation check

The manipulation check was performed to identify changes in the emotional states induced by the picture stimuli. The self-assessment manikin (SAM) was administered in a paper-and-pencil format in this study. It was used to measure the pleasure (valence) and arousal associated with the four picture categories. This is a questionnaire with a 9-point scale. Participants were asked to rate the valence of the pictures on a scale ranging from one (very negative) to nine (very positive) and the arousal level of the pictures on a scale ranging from one (not arousing) to nine (strongly arousing). Clear instructions were provided to the patients, requesting them to indicate their perceived positivity/negativity and arousal associated with the pictures.

### 2.8 Statistical analysis

The analysis of demographic variables was done using descriptive statistics, which included measures such as means, SDs, frequencies, and percentages. A one-way analysis of variance (ANOVA) was employed to assess the valence and arousal ratings for the pictures. Bonferroni tests were conducted as post-hoc analyses to explore and compare specific differences between groups. To evaluate the effects of viewing the picture categories on mood states and suicidal ideations, two-way repeated measures ANOVAs for eight dimensions of the Brunel Mood Scale (BRUMS) and the Beck Scale for Suicidal Ideation (BSSI) were performed with the within factors of the picture categories (4 categories; loved ones, strangers, natural landscapes, optical illusions) and the between factors of time (2 times; before and after viewing the pictures). Contrasts were utilized to dissect and analyze the main effects and interactions in a more detailed manner [48]. The effect size was estimated by η^2^ and Cohen's d. Consistent with Cohen's recommendations, the effect sizes were categorized as small (d = 0.2), medium (d = 0.5), and large (d = 0.8) to provide a meaningful interpretation of the magnitude of the effects observed [49].

## 3 Results

Out of the 85 patients who met the criteria for participation and indicated their willingness to take part in the study, a total of 78 individuals (91.76 %) successfully completed all the study requirements. A subset of seven patients had to be excluded from the study for specific reasons. Four patients were excluded as they did not provide the required photographs of their loved ones, while the remaining patients were excluded due to being discharged before the completion of the study sessions.

The average age of the participants was 37.34 years old [standard deviation (SD) = 12.12]. Most patients were female (67.9 %), married (73.1 %), and about 43.6 % of them had primary or secondary education. On average, the patients reported a history of depressive symptoms lasting 4.84 months (SD = 4.01). The patients, on average, spent a duration of 14.51 (SD = 7.38) days in the hospital before their first study session.

### 3.1 Manipulation check ratings

The results of the analysis of variance (ANOVA) indicated significant differences among the picture categories in terms of valence rating (F (3,311) = 26.88, p < 0.001) and arousal rating (F (3,311) = 11.28, p < 0.001). Post-hoc tests were conducted to further explore these differences. The findings revealed that photographs of loved ones received the highest ratings for pleasantness, significantly higher than photographs of landscapes (p < 0.001), strangers (p < 0.001), and optical illusions (p < 0.001). Moreover, photographs of landscapes were rated more positively than photographs of strangers (p < 0.001) and optical illusions (p < 0.001). There were no significant differences in valence ratings between photographs of strangers and optical illusions. In terms of arousal ratings, post hoc tests indicated that photographs of loved ones, landscapes, and optical illusions were similarly rated as arousing, while they were significantly different from photographs of strangers (compared to loved ones: p < 0.001; compared to landscapes: p < 0.004; compared to optical illusions: p < 0.004). For specific means and standard deviations, please refer to Table 1.

**Table 1:** Valence and arousal ratings for the 4 categories of pictures presented to the depressive patients.

Pictures									
		LOVED ONES		STRANGERS		LANDSCAPES		OPTICAL ILLUSIONS	
Variable		Mean	SD	Mean	SD	Mean	SD	Mean	SD
Valence		7.44	0.63	5.64	1.92	6.55	1.18	5.66	1.75
Arousal		6.23	0.99	4.97	1.76	5.73	1.13	5.71	1.42

Abbreviations: SD, standard deviation.

### 3.2 Effects of viewing the pictures on mood states and suicidal ideation

Table 2 displays Means and SDs for mood states and suicidal ideation.

**Table 2:** Repeated Measures ANOVAs, Means ± SDs and effect sizes (Cohen's d).

Measure		Tension	Depression	Anger	Fatigue	Confusion	Vigor	Calmness	Happiness	Suicidal ideation
Loved ones' photographs	Pre-test M±SD	10.83 ± 3.13	11.78 ± 1.81	8.84 ± 4.08	9.73 ± 3.60	9.00 ± 4.91	5.11 ± 2.33	3.06 ± 2.35	5.08 ± 4.10	10.35 ± 7.94
	Post-test M±SD	8.41 ± 3.29	8.32 ± 2.11	8.62 ± 4.06	6.16 ± 3.69	9.17 ± 4.83	8.73 ± 2.70	6.43 ± 2.84	9.98 ± 3.15	9.55 ± 7.84
	Effect size, d	0.75	1.76	0.05	0.97	0.03	1.43	1.29	1.34	0.10
**Strangers' photographs**	Pre-test M±SD	11.06 ± 3.81	11.50 ± 2.58	8.10 ± 4.51	9.30 ± 4.80	9.41 ± 4.57	5.74 ± 2.85	3.23 ± 2.31	6.61 ± 2.93	10.85 ± 8.96
	Post-test M±SD	11.25 ± 3.64	11.48 ± 2.47	8.08 ± 4.49	8.33 ± 4.43	9.39 ± 4.56	5.80 ± 2.80	3.33 ± 2.35	6.76 ± 3.10	9.84 ± 8.48
	Effect size, d	0.05	0.008	0.004	0.21	0.004	0.02	0.04	0.05	0.11
**Landscape photographs**	Pre-test M±SD	10.43 ± 3.22	10.98 ± 2.50	9.26 ± 3.34	9.96 ± 4.15	9.03 ± 4.53	4.93 ± 3.43	3.48 ± 3.06	7.03 ± 2.94	10.64 ± 8.91
	Post-test M±SD	8.44 ± 2.93	10.73 ± 2.79	9.24 ± 3.46	7.62 ± 4.09	8.94 ± 4.62	7.20 ± 4.61	6.12 ± 3.49	7.97 ± 2.88	11.14 ± 14.40
	Effect size, d	0.64	0.09	0.006	0.56	0.02	0.55	0.80	0.32	0.04
**Optical illusions**	Pre-test M±SD	11.06 ± 3.31	10.96 ± 2.69	8.48 ± 4.49	9.11 ± 5.17	8.52 ± 5.19	5.75 ± 3.36	2.91 ± 2.73	6.74 ± 3.27	12.19 ± 14.02
	Post-test M±SD	11.17 ± 3.30	11.03 ± 2.75	8.60 ± 4.06	8.50 ± 5.81	8.53 ± 5.20	5.58 ± 3.15	2.82 ± 2.64	6.87 ± 3.13	11.39 ± 13.12
	Effect size, d	0.03	0.02	0.02	0.11	0.002	0.05	0.03	0.04	0.05

Abbreviations: M, mean; SD, standard deviation.

NOTE: Tension, depression, anger, fatigue, confusion, vigor, calmness, and happiness were measured with the Brunel Mood Scale (BRUMS), and Suicidal ideation was evaluated using the Beck Scale for Suicide Ideation (BSSI).

The results of the repeated measures ANOVAs indicated significant main effects of the picture category on ratings of tension (F (1, 308) = 6.39, P < 0.001), depression (F (1, 308) = 4.72, P = 0.003), and calmness (F (1, 308) = 11.0, P < 0.001), but not on ratings of anger (F (1, 308) = 1.06, P = 0.36), fatigue (F (1, 308) = 0.75, P = 0.51), confusion (F (1, 308) = 0.44, P = 0.72), vigor (F (1, 308) = 2.57, P = 0.05), happiness (F (1, 308) = 1.69, P = 0.16) and suicidal ideation (F (1, 308) = 0.45, P = 0.71). These findings suggest that, when disregarding the effects of time, there were significant differences among the picture categories in terms of the average levels of tension, depression, and calmness. As a result, contrast analysis conducted to compare the picture categories yielded statistically significant differences between photographs of loved ones (vs. strangers: P = 0.02, vs. optical illusions: P = 0.02) and landscapes (vs. strangers: P = 0.007, vs. optical illusions: P = 0.008) with photographs of strangers and optical illusions in terms of the marginal mean of tension. Also, a significant difference was identified between photographs depicting loved ones and those featuring strangers (P = 0.002) regarding the marginal mean of depression. Moreover, significant differences between photographs of loved ones (vs. strangers: P = 0.001, vs. optical illusions: P < 0.001) and landscapes (vs. strangers: P < 0.001, vs. optical illusions: P < 0.001) with photographs of strangers and optical illusions regarding mean score of calmness were found (Table 3).

**Table 3:** Analyses of variance (repeated measures design): F-ratios, p-values, partial η^2^.

*Measure*	df	F	P	η^2^
**Picture category**				
**Tension**	1308	6.39	<0.001	0.05
**Depression**	1308	4.72	0.003	0.04
**Anger**	1308	1.06	0.36	0.01
**Fatigue**	1308	0.75	0.51	0.007
**Confusion**	1308	0.44	0.72	0.004
**Vigor**	1308	2.57	0.05	0.02
**Calmness**	1308	11.0	<0.001	0.09
**Happiness**	1308	1.69	0.16	0.01
**Suicidal ideation**	1308	0.45	0.71	0.004
**Time**				
**Tension**	1308	138.83	<0.001	0.31
**Depression**	1308	232.56	<0.001	0.43
**Anger**	1308	0.66	0.41	0.002
**Fatigue**	1308	168.94	<0.001	0.35
**Confusion**	1308	0.21	0.64	0.001
**Vigor**	1308	255.03	<0.001	0.45
**Calmness**	1308	360.01	<0.001	0.53
**Happiness**	1308	174.28	<0.001	0.36
**Suicidal ideation**	1308	2.48	0.11	0.008
**Time × Group**				
**Tension**	3308	62.28	<0.001	0.37
**Depression**	3308	202.45	<0.001	0.66
**Anger**	3308	2.51	0.05	0.02
**Fatigue**	3308	21.94	<0.001	0.17
**Confusion**	3308	1.39	0.24	0.01
**Vigor**	3308	100.63	<0.001	0.49
**Calmness**	3308	122.76	<0.001	0.54
**Happiness**	3308	96.63	<0.001	0.48
**Suicidal ideation**	3308	1.06	0.36	0.01

Abbreviations: df, degrees of freedom.

There was also a significant main effect of time on ratings of tension (F (1, 308) = 138.83, P < 0.001), depression (F (1, 308) = 232.56, P < 0.001), fatigue (F (1, 308) = 168.94, P < 0.001), vigor (F (1, 308) = 255.03, P < 0.001), calmness (F (1, 308) = 360.01, P < 0.001), and happiness (F (1, 308) = 174.28, P < 0.001). There was not a significant main effect of time on ratings of anger (F (1, 308) = 0.66, P = 0.41), confusion (F (1, 308) = 0.21, P = 0.64) and suicidal ideation (F (1, 308) = 2.48, P = 0.11).

Similarly, the interaction of picture category × time for anger (F (3, 308) = 2.51, P = 0.05), confusion (F (3, 308) = 1.39, P = 0.24) and suicidal ideation (F (3, 308) = 1.06, P = 0.36) was not significant (see Table 3 for more detail). This shows that none of the picture categories resulted in a significant reduction in anger, confusion, and suicidal ideation from the initial assessment to the subsequent evaluation conducted after viewing the pictures.

With regard to tension (F (3,308) = 62.28, P < 0.001), depression (F (3,308) = 202.45, P < 0.001), fatigue (F (3,308) = 21.94, P < 0.001), vigor (F (3,308) = 100.63, P < 0.001), calmness (F (3,308) = 122.76, P < 0.001), and happiness (F (3,308) = 96.63, P < 0.001), significant interactions of picture category × time were found (Table 3). With regard to tension, contrast analysis revealed significant interactions when photographs of loved ones (vs. strangers: P < 0.001, vs. optical illusions: P < 0.001) and landscapes (vs. strangers: P < 0.001, vs. optical illusions: P < 0.001) were compared with those of strangers and optical illusions. It means that there was a greater reduction in tension after viewing the photographs of loved ones and landscapes in comparison to the other categories of pictures. Moreover, a significant interaction was identified concerning depression when comparing photographs of loved ones with those of strangers (P < 0.001), landscapes (P < 0.001), and optical illusions (P < 0.001). This finding suggests that there was a greater reduction in depression following the viewing of loved ones' photographs compared to the other picture categories. The most significant reduction in fatigue was observed after viewing photographs of loved ones in comparison to the other categories of pictures (vs. landscapes: P < 0.003, vs. strangers: P < 0.001, vs. optical illusions: P < 0.001). Also, photographs of landscapes led to a reduction in fatigue more than photographs of strangers (P < 0.001) and optical illusions (P < 0.001). With regard to vigor, significant interactions were found when comparing photographs of loved ones with the photographs of strangers (P < 0.02) and optical illusions (P < 0.01). No significant interaction was shown regarding vigor when comparing photographs of loved ones with photographs of landscapes. It indicates that photographs of loved ones similar to landscapes lead to an enhanced vigor more than photographs of strangers and optical illusions. Photographs of loved ones increased calmness (vs. strangers: P < 0.001, vs. optical illusions: P < 0.001, vs. landscapes: P < 0.001) and happiness (vs. strangers: P < 0.001, vs. optical illusions: P < 0.001, vs. landscapes: P < 0.001) more than the other picture categories. Also, photographs of landscapes led to an enhancement of calmness (vs. strangers: P < 0.001, vs. optical illusions: P < 0.001) and happiness (vs. strangers: P < 0.01, vs. optical illusions: P < 0.01) more than photographs of strangers and optical illusions.

## 4 Discussion

Mental imagery has been relatively overlooked in the assessment and treatment of depression and suicidal ideation. There is a limited body of research that has focused on the beneficial effects of positive mental imagery on clinical symptoms in the context of clinical depression [50,51] and especially suicidal ideation [20]. In the current study, we applied a well-established positive imagery procedure based on the presentation of loved ones’ photos to depressive suicidal patients. Using the photographs of loved ones, we showed that the imagery for past- or future-directed positive events had beneficial effects on mood states, especially tension, depression, fatigue, vigor, calmness, and happiness, in depressed suicidal patients. To our knowledge, this is the first time that such an effect has been shown for depressive suicidal patients.

In line with our expectations, the findings showed a significant increase in vigor, happiness, and calmness after viewing the photographs of loved ones compared to photos of strangers and optical illusions. Our results are consistent with those of Werner-Seidler & Moulds, who found that depressive patients who focused on the concrete, sensory aspects of their pleasant memories could emotionally benefit from the recall of their positive memories [16]. Pictures can serve as potent triggers for recalling memories and the emotions tied to them, owing to their remarkable resemblance to actual event experiences [52]. However, there are no studies examining the effects of mental imagery using loved ones' photographs on depressive suicidal patients. Therefore, relatively relevant studies in this field must be mentioned. In a recent investigation carried out by Fernández-Pérez, Ros, and Latorre (2023), the efficacy of diverse image categories, encompassing personal photographs, images of specific places, and those from the International Affective Picture System (IAPS), in evoking positive autobiographical memories has been demonstrated as a method for inducing positive moods and promoting mood recovery in both healthy older adults and young individuals [53]. Based on the findings of this research, all three categories of images demonstrated equal effectiveness in enhancing positive emotions [53]. However, it is worth mentioning that in this study, participants chose personal photographs from their private collections, whereas images of specific places were sourced from the internet and held personal significance, such as the participant's hometown, current city of residence, or locations visited during trips, etc. Furthermore, there are other differences between this study and the present study, including the nature of groups of photographs, the study population, the difference in methodology, and the use of imagery for past- or future-oriented positive events related to the photographs of loved ones in the present study. These can lead to some differences between the results of these two studies.

There are several possible explanations for the beneficial effects of positive mental imagery of autobiographical memories using the photographs of loved ones in depressive suicidal patients. Research has demonstrated that individuals who have experienced major depression and attempted suicide often exhibit a diminished specificity in their autobiographical memories. This reduced specificity in recalling personal memories serves as a common vulnerability factor, potentially contributing to the development of affective disorders and suicide attempts [54]. The capacity to access specific autobiographical memories serves as a significant indicator of emotional well-being in individuals [55]. The utilization of visual stimuli to evoke positive emotions from events surpasses the effects of verbal processing, leading to an enhanced mood state [56]. Thus, in this study, the utilization of photographs featuring loved ones appears to closely align with the sensory experience of past events, eliciting the recall of positive emotions. It seems that by having mental imagery related to loved ones photographs in this study, we utilized the effective role of patients' supportive systems and constructive family relationships in improving depressive suicidal patients [57]. Individuals feel attachment-induced safety and security when they are with significant others. The presence of attachment figures can have positive effects on individuals' life expectancy and psychological resilience [58,59]. Furthermore, at the psychophysiological level, research has demonstrated that observing the faces of familiar loved ones triggers a profoundly positive emotional response that cannot be solely attributed to familiarity or arousal [60,61]. These findings potentially challenge the notion that the positive effects of social support solely arise from direct supportive interactions. They suggest that even simple reminders of loved ones, such as looking at photographs, can be enough to evoke positive feelings of support, security, vigor, happiness, and calmness.

In the present study, the patients were asked to explain the good memories evoked by the shown photos. They were also requested to describe the good characteristics of their loved one, imagine themselves with him/her in a good past or future positive event, and explain it in detail. In their recent research conducted with a non-clinical sample, Hallford et al. (2020) examined the influence of directed episodic thinking on predicted anticipatory pleasure, specifically focusing on past or future pleasant events. The participants were instructed to deliberately concentrate on the positive aspects of previous or anticipated events, adopting a first-person perspective. The overall findings of this study provide empirical support for their hypothesis that directed episodic thinking focused on pleasant events, whether related to the past or the future, enhances predicted anticipatory pleasure compared to baseline ratings [62]. In addition, Renner et al. (2021), in their recent valuable study, proposed a conceptual model of prospective mental imagery. This model suggests that envisioning positive future events through mental imagery can enhance reward anticipation, motivation, and engagement in rewarding activities. This process may lead to a reduction in depressive symptoms by promoting positive emotions and increasing involvement in rewarding experiences [50]. In this study, imagery for past- or future-oriented positive events related to the loved ones picture may enhance the pleasure and motivation for life and emotional wellbeing in patients. Furthermore, cognitive models of depression, such as Beck's cognitive theory, emphasize the preponderance of negative mood-congruent memories and the tendency to selectively recall negative information in depression, positing that people with major depressive disorder have negative biases in attention, memory, and interpretation [63,64]. Several studies have also suggested depressed patients are biased toward negative stimuli and reduce attention to positive emotional stimuli [65,66]. In this regard, it is crucial to acknowledge that autobiographical memories (AMs), which significantly impact self-perception [67], are prone to being influenced by negative schemas in individuals experiencing depression. Studies have indicated that positive AMs in individuals with major depressive disorder (MDD) often exhibit reduced vividness [68], a higher likelihood of being recalled from an observer perspective rather than as an active participant [69], and are associated with more distant past events [70]. Moreover, individuals with elevated depressive symptoms tend to recall positive memories less frequently [71] and with slower retrieval rates [72]. It seems that showing positive imagery using the photographs of loved ones in the present study might increase the focus of depressive suicidal patients on positive mood-congruent memories. Furthermore, viewing the loved ones picture besides the imagery for past- or future-oriented positive events has helped depressive suicidal patients recall positive autobiographical memories easier, closer, and more vividly from a first-person perspective. This matter has led to positive emotions in the participating patients and improved vigor, happiness, and calmness. Hence, future-oriented mental imagery holds promise as a potential experimental intervention in the treatment of individuals with depression and suicidal tendencies. Additionally, our results contradict the arguments offered by Werner-Seidler & Moulds that distraction may be effective in mood repair experienced by depressive patients [16], because we found no evidence that viewing strangers' photographs and optical illusions (considered non-emotional distraction tasks) can significantly improve mood states. The high rating of valence for loved ones' photographs compared to the other picture categories confirms the positive effect of this type of stimulus on patients with depression [29]. In sum, our findings suggest that viewing the photographs of loved ones might improve the mood states of depressed individuals. However, more studies are needed to investigate how viewing photos of loved ones may increase positive moods in this population.

According to the results, the patients experienced a significant reduction in fatigue and tension and an increase in vigor, happiness, and calmness after viewing photographs of landscapes compared to strangers and optical illusions' pictures. Based on the findings from Fernández-Pérez, Ros, and Latorre's study (2023), it was observed that images of specific places, including those from the International Affective Picture System (IAPS), as well as personal photographs, were successful in enhancing positive affect. Interestingly, when it came to reducing negative affect, personal photographs had the most significant impact among young adults, whereas in the older age group, the IAPS images were the most effective [56]. It is important to note that in the aforementioned study, the images of places used in the intervention were obtained from the Internet and were specifically associated with meaningful and significant locations in each individual's life. Differentials in the nature of photograph categories and study methodology can be an important reason for the differentials between the results of this study and the present study. Jo et al. (2019), in their recent systematic review of 37 studies, have shown the improvement of tension and fatigue and the relaxing effects of viewing images of natural environments using various objective physiological measurement indicators [73]. The authors concluded that contact with nature (mainly through display stimuli such as photographs, 3D images, virtual reality, and landscape videos) leads to physiological relaxation and stress relief [73]. Based on Attention Restoration Theory (ART), nature is considered a positive distraction [74]. According to this theory, nature draws attention away from negative stimuli such as stress [75]. It is probably that the innate tendency toward natural landscapes and distraction from depressive symptoms may lead to reduced fatigue and tension as well as increased calmness and happiness in depressed patients. It seems that such resources might be a relatively easy and inexpensive tool to create happiness, calmness, and vigor in hospitalized patients with depression [76].

We did not find the positive impact of viewing the photographs on suicidal ideation in depressive suicidal patients. Prior research in the domain of employing mental imagery for individuals experiencing suicidal ideation primarily focused on assessing the impact of mental images related to suicide [12,[17], [18], [19]]. However, Knagg et al.'s study (2022) [20] stands out as the sole study demonstrating promising indications regarding the feasibility and acceptance of positive mental imagery interventions among students grappling with suicidal thoughts [20]. It is worth highlighting that this study utilized a brief relaxation technique followed by guided visualization of a positive memory [20], differing from the positive mental imagery approach employed in the present research. The disparity observed between the findings of the present study and the previous study could potentially be attributed to variations in the mental imagery intervention and methodological approaches employed in both studies.

Individuals with a suicidal history often exhibit overgeneralized autobiographical memories, which lack specificity and detail and are linked to undesirable outcomes such as impaired interpersonal problem-solving and a sense of hopelessness about the future [77]. This tendency might have contributed to the absence of a positive impact of viewing photographs on suicidal ideation among the participants in this study. On the other hand, suicidal ideation might arise as a result of a series of unpleasant factors that threaten an individual's stability. Many models and theories, such as the Integrated Motivational-Volitional Model of Suicidal Behavior (IMV model) [78], the Stress-Diathesis Model [79], and the Behavioral-Cognitive Theory [80], have pointed to the multifactorial feature of suicide. The Integrated Motivational-Volitional Model (IMV) delves deep into the multifactorial nature of suicide. By dissecting biopsychosocial factors and core constructs like defeat and suicidal ideation, it highlights the intricate web of influences leading to suicidal behavior. Lower-order moderators further enrich its complexity, revealing the nuanced interplay of factors in suicidal tendencies [78]. The Stress-Diathesis Model posits that suicidal ideation and behavior arise from a multifaceted interplay of biological and psychological risk factors, as well as individual experiences and stressors [79,81]. The behavioral-cognitive theory has also proposed three constructs resulting in suicidal behavior, including dispositional vulnerability, cognitive processes related to psychiatric disturbance, and cognitive processes associated with suicidal acts [80]. So, it seems that the mental imagery used in this study couldn't involve all factors related to suicidal ideation and behavior, and it has led to a lack of positive impact of viewing the photographs on suicidal ideation in the participants. It demonstrates that, rather than regulating suicidal ideation to the domain of a symptom, clinicians should situate such events within the complexity of each individual [82]. It seems that the study of suicidal ideation using approaches such as phenomenology (compared to quantitative approaches) may better depict the real results of the interventions targeting suicide prevention.

While the current study yielded promising results, it is important to acknowledge several limitations. First, the emotional state induced by photographs of loved ones may vary depending on the quality of the relationship. Another limitation of the study is that it excludes patients with severe depression. Besides, the patients participating in the study were treated for depression for two weeks on average at the study time. It is suggested to assess the effects of viewing the pictures on a broader range of patients with depression, including those with severe depression, and at different time intervals of hospitalization. Another important limitation of our study is that the Beck Scale for Suicidal Ideation (BSSI), which was used to assess suicide ideation, may not capture moment-to-moment changes due to its focus on reflecting on ideation over an unspecified time period. Future research should consider employing alternative methodologies that can measure real-time and dynamic shifts in suicide ideation, allowing for a more nuanced understanding of the phenomenon. This may involve the development of new measurement tools or the adaptation of existing ones to better align with the complexity and variability of suicide ideation. The effect of cultural context on emotions and emotion regulation may impact valence, arousal as well as mood states evoked by images. Future studies could explore the impact of cultural context on the effect of loved ones' pictures on mood states. Another limitation of this study is not considering the vividness of the visualized images in the patient's memory. Although the evaluation of the valence and arousal of images can reflect the effectiveness of the visualized images in the patient's memory to some extent, it is recommended that this be specifically addressed in future studies.

## 5 Conclusions

Overall, our positive imagery procedure using the presentation of loved ones’ photos, newly developed by our group, showed beneficial effects on the mood states of depressive suicidal patients but not on their suicidal ideation. Incorporating more focus on positive imagery, particularly through the use of loved ones' photographs, may offer distinct advantages and present new opportunities for the treatment of depressed mood.

The current results can be used in mental health nursing, psychotherapy, and psychiatry. In this regard, positive imagery using photographs of loved ones may be considered along with other non-pharmaceutical strategies to induce a positive mood in hospitalized patients with major depression. However, comprehensive studies are needed to assess the effect of loved ones photographs on suicidal ideation by considering different related factors. Furthermore, it is suggested to study the long-term effects of positive mental imagery on mood and suicidal ideation in a broader range of patients with depression. The results also provide clear direction for future experiments on the neural and cognitive mechanisms that underlie the relationship between depression and mental imagery. Further studies are needed to examine how and for whom positive mental imagery works. Such knowledge may aid future therapeutic innovation in the context of depression.

## Funding

This study was funded by the 10.13039/501100004320Shiraz University of Medical Sciences (Grant number: 13115).

## Data availability statement

The data associated with this study has not been deposited in a publicly available repository. The datasets utilized and/or analyzed during the present study can be obtained from the corresponding author upon reasonable request.

## Ethics approval and consent to participate

The study received ethical approval from the local Ethics Committee of Shiraz University of Medical Sciences (IR.SUMS.REC.1399.011). All eligible participants were provided with comprehensive information about the study's objective and the voluntary nature of their involvement. The study procedures adhered to the ethical standards set by the local Ethics Committee of Shiraz University of Medical Sciences and were in accordance with the Helsinki Declaration of 1975, as revised in 2000. All patients included in the study provided informed consent prior to their participation.

## Consent for publication

Not applicable.

## CRediT authorship contribution statement

**Fahimeh Alsadat Hosseini:** Writing – review & editing, Writing – original draft, Project administration, Investigation, Data curation. **Maryam Shaygan:** Writing – original draft, Methodology, Investigation, Funding acquisition, Formal analysis, Conceptualization. **Zahra Jahandideh:** Project administration, Investigation, Data curation.

## Declaration of competing interest

The authors declare that they have no known competing financial interests or personal relationships that could have appeared to influence the work reported in this paper.

## List of abbreviations

BRUMS: Brunel Mood Scale
BSSI: Beck Scale for Suicide Ideation
MDD: Major Depressive Disorder
DSM-V: Diagnostic and Statistical Manual of Mental Disorders, Fifth Edition
SCID-5: Structured Clinical Interview for DSM
MDC: Minimal Difference Change
SI: Suicidal Ideation
SAM: Self-Assessment Manikin
ART: Attention Restoration Theory
CBM: Cognitive Bias Modification
